# Genotype‐Specific Small EVs Released by *Giardia lamblia* Act as Mediators of Phenotypic Adaptation Under Metronidazole‐Induced Stress

**DOI:** 10.1002/jev2.70139

**Published:** 2025-09-01

**Authors:** Gabriel Luna Pizarro, Jerónimo Laiolo, Nehuén Salas, Rocío G. Patolsky, Luciano Díaz Pérez, Camilo Cotelo, Constanza Feliziani, Andrea Silvana Rópolo, María Carolina Touz

**Affiliations:** ^1^ Instituto de Investigación Médica Mercedes y Martín Ferreyra, Consejo Nacional de Investigaciones Científicas y Técnicas (INIMEC‐CONICET) Universidad Nacional de Córdoba Córdoba Argentina; ^2^ Universidad Católica de Córdoba Córdoba Argentina

**Keywords:** drug‐resistance mechanisms, *Giardia lamblia*, metronidazole sensitivity, phenotypic adaptation, small extracellular vesicles (sEVs)

## Abstract

*Giardia lamblia*, a eukaryotic intestinal parasite, produces small extracellular vesicles (sEVs) as a conserved evolutionary mechanism. This study investigates the functional role of sEVs in modulating drug response traits among *G. lamblia* parasites. Here, we showed that sEVs derived from metronidazole (MTZ)–resistant clones modify the expression of enzymes involved in MTZ metabolism and the production of reactive oxygen species (ROS) in recipient wild type parasites. The transfer efficiency and phenotypic impact vary depending on the genetic background of the isolates, highlighting a genotype‐specific mechanism. Our findings reveal that sEVs act as mediators of phenotypic adaptation in *G. lamblia*, enhancing parasite survival under drug‐induced stress. This study highlights the significance of sEVs in drug‐sensitive dynamics and lays the groundwork for investigating therapeutic interventions that target EV‐mediated sensitivity in giardiasis.

## Graphical abstract

Small extracellular vesicles (RsEVs) derived from metronidazole‐resistant *Giardia lamblia* clones (WB/1267_MTZ_r and GS/M_MTZ_r) modulated drug sensitivity in wildtype parasites by altering reactive oxygen species (ROS) levels and the expression of MTZ‐metabolising enzymes in a genotype‐dependent manner. These findings highlight RsEVs as key mediators of phenotypic adaptation and promising targets for novel anti‐giardial therapies.

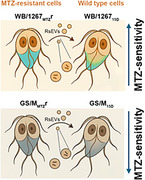

## 1 Introduction

Giardiasis, caused by the protozoan *Giardia lamblia* (syn. *G. intestinalis* or *G. duodenalis*), is the most common non‐viral and non‐bacterial diarrheal illness worldwide, leading to significant health issues such as weight loss, malnutrition, growth delays in children, delayed puberty, impaired cognitive development and even premature death (Farthing 1997; Adam 2021). The primary treatments for giardiasis are metronidazole (MTZ) and tinidazole, which belong to the 5‐nitroimidazole (5‐NI) family. However, persistent infections can occur due to reinfection, inadequate drug dosage, immunosuppression, drug‐resistant strains and sequestration of *Giardia* in the gallbladder or pancreatic duct. Current treatments for parasitic infections are systemic, require long‐term medication, come with side effects and are ineffective against resistant strains. MTZ resistance in *Giardia* poses a significant challenge in treating giardiasis, and resistance has been observed across different medications (Gardner and Hill 2001; Arguello‐Garcia et al. 2020).

According to a widely accepted model, nitro compounds are activated by reduction, producing toxic intermediates that cause oxidative stress (Lloyd et al. 2003; Muller et al. 2007). MTZ is a prodrug, pharmacologically inactive in its original form, which requires metabolic activation within the cell to become its active form. Understanding the causes of MTZ resistance involves exploring the activation of nitro compounds and the subsequent detoxification pathways within the parasite. While resistance to nitro compounds is commonly observed both in vitro and in vivo, fresh resistant patient isolates are challenging to maintain in axenic culture. Consequently, most studies rely on generating resistant model strains in vitro and comparing them to isogenic wild type strains (Upcroft 1998). In this sense, transcriptional changes and proteome analysis have highlighted significant differences in gene expression between susceptible and resistant genotype A, subtype AI strain (Gardner and Hill 2001; Arguello‐Garcia et al. 2020; Krakovka et al. 2022). Our working hypothesis is that strain genotypes can influence these changes, complicating the identification of common and specific resistance patterns across studies.

*Giardia lamblia* isolates infecting humans are primarily classified into two genetically distinct assemblages, A and B, which differ in host specificity, genetic diversity, and clinical presentation. Assemblage A is subdivided into subtypes AI, AII and AIII: AI is found in both humans and animals, indicating zoonotic transmission; AII is predominantly human‐specific; and AIII is mainly isolated from wild animals. Although less clearly structured, Assemblage B includes subtypes BIII and BIV, which are commonly found in humans. Assemblage B displays greater genetic variability than A and is often associated with more pronounced small intestinal inflammation, including villous shortening/atrophy and lamina propria inflammation, compared to genotype A (Jerlstrom‐Hultqvist et al. 2011; Franzen et al. 2009, reviewed in Caccio et al. 2018; Arguello‐Garcia and Ortega‐Pierres 2021). Furthermore, genotype B isolates induce more significant alterations in the intestinal mucosa and a reduction in the enzymatic activity of the brush border than genotype A (Homan and Mank 2001). Additionally, genotype B is more resistant to reactive oxygen species (ROS) and nitric oxide (NO) (Thomas and Gwenin 2021; Eckmann et al. 2000; Stadelmann et al. 2013; Ansell et al. 2015).

Extracellular vesicles (EVs) are membrane‐bound particles released into the extracellular environment, mediating communication and information transfer between cells and organisms across all domains of life (Gill et al. 2019). These vesicles transport molecular cargo, including cytosolic and membrane proteins, lipids and RNA (Gruenberg and Stenmark 2004; Hurley 2008; Colombo et al. 2014). EVs play significant roles in normal physiology and pathogen‐host interactions, spreading antigens and infectious agents. Initially studied for their functions in immune surveillance, EVs also facilitate various modes of communication between parasites (Marcilla et al. 2014; Montaner et al. 2014; Szempruch et al. 2016; Mantel et al. 2016; Mantel et al. 2013). So far, studies on small and large EVs have shown that they mainly engage in cellular stress responses and contribute to the resistance against chemotherapeutic drugs in eukaryotic cells, and have also been recently reported in *Leishmania* (O'neill et al. 2019; Douanne et al. 2022). *Giardia* trophozoites produce different populations of EVs under various environmental conditions or biological stimuli, including large extracellular vesicles (mainly enriched in microvesicles—MVs) and small extracellular vesicles (sEVs, exosome‐like) or the complete secretome (EVs plus free secreted proteins) (Evans‐Osses et al. 2017; Ma'ayeh et al. 2017; Dubourg et al. 2018). Our research group has previously examined the formation and release of exosomal‐like vesicles (ElVs), now called sEVs, in *Giardia* trophozoites and compared the types and quantities of small RNAs (sRNAs) in sEVs from strains with different genotypes (Natali et al. 2023; Moyano et al. 2019).

Drug sensitivity, resistance and tolerance are fundamental concepts for understanding microbial responses to antimicrobial agents such as antibiotics. Drug sensitivity describes the degree to which a microorganism is inhibited or killed by a drug. Sensitivity is a continuous and quantifiable trait, typically measured by IC_50_ in dose–response assays. It reflects the integrated effect of drug uptake, activation, detoxification and stress response pathways, and can be modulated by external factors without involving permanent genetic changes (Leitsch 2015; Ansell et al. 2015). In contrast, drug resistance is defined as the ability of microorganisms to grow and proliferate despite the presence of an antimicrobial agent, often resulting from stable genetic mutations or the acquisition of resistance genes (Blair et al. 2015; Munita and Arias 2016). Drug tolerance, unlike resistance, does not involve an increase in IC_50_. Instead, it describes a transient phenotypic adaptation that allows a subpopulation of microorganisms to survive exposure to high drug concentrations without acquiring genetic resistance, often by entering a dormant or slowed metabolic state (Brauner et al. 2016).

In this work, we explored whether small extracellular vesicles produced by MTZ‐resistant clones (RsEVs) could influence drug response phenotypes within and between genotypes A and B of *G. lamblia*. This study provides, for the first time, evidence that RsEVs can induce changes in the expression of enzymes involved in metronidazole (MTZ) metabolism and reactive oxygen species (ROS) production, and that these changes depend on the molecular content of the RsEVs and the genotypic background of the recipient wild type parasites. This vesicle‐mediated modulation facilitates the rapid emergence of subpopulations with differential sensitivity to MTZ, enabling swift adaptation to drug‐induced stress. Such genotype‐dependent responses may enhance parasite survival under fluctuating environmental conditions and represent an early stage in the development of stable resistance.

## 2 Materials and Methods

### 2.1 Cell Lines and Cell Culture

Axenic cultures of *Giardia* trophozoites of isolates WB clone 1267 (ATCC 50582, Assemblage A, subtype AI) and GS/M (ATCC 50581, Assemblage B, subtype IV) were purchased at American Type Culture Collection (www.atcc.org, accessed on 1 March 2008). Trophozoites were routinely grown in 16 mL screw‐cap tubes (NuncTM, ThermoFisher Scientific, Waltham, MA, USA) in TYI‐S33 medium, supplemented with 10% adult bovine serum (Euroclone, Pero, Italy) and bovine bile (Sigma‐Aldrich S.R.L., Milan, Italy) (Keister 1983) at 37°C. Log‐phase cultures were harvested after cooling the culture vials on ice for 15 min and centrifugation at 700 × *g* for 10 min.

### 2.2 Production of Metronidazole‐Resistant *Giardia* Clones

Metronidazole (MTZ)–resistant *Giardia* clones were generated by gradually exposing will type trophozoites to increasing sublethal concentrations of MTZ, followed by selection and subcloning (Arguello‐Garcia et al. 2009). Briefly, *Giardia* trophozoites from strains WB/1267 (ATCC 50582, Assemblage A) and GS/M (ATCC 50581, Assemblage BIV) were cultured in complete growth medium supplemented with sublethal concentrations of MTZ (Cat. No. M3761, Sigma‐Aldrich). The adaptation process involved a stepwise protocol, beginning with an initial sublethal concentration of 0.25 µM of MTZ and progressing through successive rounds of culture in higher concentrations up to 6.4 and 10.5 µM for strains WB/1267_MTZ_r and GS/M_MTZ_r, respectively. At each stage, adapted trophozoites were selected and continuously cultured under these conditions for 730 days, with regular monitoring to assess cell viability.

### 2.3 Cell Viability Assay

To assess the cytotoxic effect of MTZ, the 3‐(4,5‐dimethylthiazol‐2‐yl)‐2,5‐diphenyltetrazolium bromide (MTT) colorimetric assay was performed as previously described (Barzola et al. 2024; Garcia‐Bustos et al. 2025). Briefly, trophozoites from WB/1267, GS/M, WB/1267_MTZ_r, GS/M_MTZ_r, WB/1267_R15_ and GS/M_R15_ isolates were seeded at a density of 5 × 10⁵ cells per well in 150 µL of complete growth medium in 96‐well plates. An additional 150 µL of medium containing serial dilutions of MTZ, previously dissolved in DMSO (final concentration 0.5% v/v, as this concentration showed no adverse effects on cell growth), was added. Following anaerobic incubation at 37°C for 48 h, the plates were centrifuged at 500 × *g* for 10 min. Subsequently, the cells were washed three times with 1 × PBS by centrifugation, and 20 µL of a 5 mg/mL MTT solution in sterile PBS was added to each well, followed by further incubation for 4 hat 37°C. After the removal of the supernatants, 100 µL of DMSO was added to solubilise the purple formazan crystals produced by metabolically viable cells. Absorbance was measured at 570 nm using a Model 680 microplate reader (Bio‐Rad, USA). Cytotoxicity percentages were calculated relative to DMSO‐treated control cells, which were considered 100% viable. The percentage of cytotoxic activity was determined using the following formula: cytotoxicity (%) = [1 − (OD of treated cells − OD of DMSO) / (OD of control cells − OD of DMSO)] × 100, where OD refers to optical density. Half‐maximal inhibitory concentrations (IC_50_), defined as the concentrations required to inhibit 50% of cell proliferation, were determined from the mean values obtained from replicate wells across three independent experiments. Resistance Fold (RF) is a measure used to quantify the amount of a drug required to inhibit a resistant microorganism compared to a sensitive (wildtype or control) strain. It is calculated using the formula: Resistance Fold (RF) = IC_50_ of resistant cells / IC_50_ of sensitive cells. RF values greater than 1 indicate increased resistance to MTZ, values less than 1 indicate reduced resistance, and values equal to 1 suggest no change in resistance.

### 2.4 RT‐qPCR Analysis

The expression of key genes involved in MTZ metabolism and reactive oxygen species detoxification was assessed using RT‐qPCR. Briefly, trophozoites from the WB/1267, GS/M, WB/1267_MTZ_r, GS/M_MTZ_r, WB/1267_R15_ and GS/M_R15_ isolates were homogenised in Trizol reagent (Cat. No. 15596026, Thermo Fisher Scientific Inc.). Total RNA was extracted using the SV Total RNA Isolation System (Cat. No. Z3100, Promega, Madison, WI, USA) following the manufacturer's protocol. Two micrograms of total RNA were reverse‐transcribed using M‐MLV Reverse Transcriptase (Cat. No. A3802, Promega). The cDNA was analysed for the pyruvate oxidoreductase (PFOR) (gene ID GL50803_114609), Nitroreductase‐1 (NR‐1) (gene ID Gl50803‐22677), superoxide reductase (SOR) (gene ID GL50803_61550) and Thioredoxin reductase (TrxR) (gene ID GL50803_9827), via real‐time PCR using SYBR Green Master Mix (Cat. No. QR100‐1KT, Thermo Fisher Scientific Inc.), with 100 ng of total RNA equivalent as single‐stranded cDNA and 800 nM of each amplification primer in a 20 µL reaction volume. Specific primers for each gene were designed using Primer Express software (Applied Biosystems, Foster City, CA, USA): PFOR Fw (CATGAACACGGAGCAGAGGT) and PFOR Rv (GAGCCCCTGAAGAACCTTCC); NR‐1 Fw (CGAGACAAAGGTAGTGGCGT) and NR‐1 Rv (CTGCCGGTGGATCTGTCTTT); SOR Fw (GAGGACCAAGGAGAAGCACG) and SOR Rv (TTGCCCTCCTTAGTGATGCC); TrxR Fw (CTCGCTGACGCCCTTATCAT) and TrxR Rv (GACACCCCCTTTTGCCAGTA). Runs were carried out on a standard 7500 system (Applied Biosystems) using a QuantStudio3 device (Applied Biosystems, Foster City, CA, USA) and QuantStudio Design & Analysis Software v1.5.2. The RT‐qPCR conditions were as follows: 50°C for 2 min, 95°C for 10 min, followed by 40 cycles of 95°C for 15 s and 60°C for 1 min. Gene expression was normalised to the housekeeping gene 18S (Fw: AAGACCGCCTCTGTCAATAA, Rv: GTTTACGGCCGGGAATACG) and calculated using the comparative ΔΔCt method. Melting curve analysis was performed to confirm the specificity of the qPCR products. The 18S rRNA gene was used as a reference gene due to its high sensitivity and specificity for *G. lamblia* qPCR assays, as previously validated by Müller et al. (2008). These assays were conducted in triplicate with duplicate reactions. All DNA oligonucleotides were purchased from Macrogen (Macrogen, Seoul, Republic of Korea).

### 2.5 Measurement of Reactive Oxygen Species (ROS)

Following the manufacturer's instructions, the Image‐iT LIVE Green Reactive Oxygen Species Detection Kit (Invitrogen, MA, USA) was used to assess intracellular ROS generation. Flow cytometry (FACS Canto II, Becton & Dickinson, NJ, USA) was employed to quantify ROS levels using the fluorescent probe 2′,7′‐dichlorodihydrofluorescein diacetate (H_2_DCFDA), which is oxidised to 2′,7′‐dichlorofluorescein (DCF) in the presence of ROS, emitting fluorescence that is proportional to the oxidative capacity of reactive species. ROS levels were measured in the following isolates: WB/1267, GS/M, WB/1267_MTZ_r, GS/M_MTZ_r, WB/1267_R15_ and GS/M_R15_, after a 48‐h treatment with 20 µM metronidazole or without the drug as a control.

### 2.6 Isolation and Purification of sEVs

Enriched sEVs were obtained using differential ultracentrifugation from the supernatant of trophozoites, as we described (Moyano et al. 2019). Briefly, 7 × 10^7^ trophozoites recovered from the monolayer were washed twice with warm PBS 1X (37°C). To avoid sEV contamination from other sources, the trophozoites were incubated in TYI‐S‐33 medium without serum and bovine bile (TYI‐S‐33/‐sbb) for 4 h at 37°C before isolation. Then, the parasites were removed by centrifugation at 1455 × *g* for 15 min, and the supernatant was recovered. After centrifugation, the supernatant was filtered through a 0.11‐µm filter (Millipore) to discard high‐size vesicles. To obtain the sEVs fraction, the filtered elution was subsequently pelleted at 100,000 × *g* for 180 min using a 60Ti rotor (Beckman‐coulter L‐70 Ultracentrifuge). The pellet was then washed with PBS and pelleted again at 100,000 × *g* in the same ultracentrifuge.

### 2.7 Identification and Characterisation of sEVs

Nanoparticle tracking analysis (NTA) was used to determine the sEVs size distribution and concentration with a ZetaView PMX‐230 Twin Laser (Particle Metrix, Germany) device according to the MISEV 2023 guidelines (Welsh et al. 2024). Briefly, sEV‐enriched samples were re‐suspended in 0.11‐µm‐filtered PBS 1X and diluted to achieve a particle concentration within the detection range (20–100 particles/frame) before measurement. Samples were manually injected into the instrument using a 1‐mL syringe. The measurements were taken at 11 different positions, with video quality set to medium and camera sensitivity set to 80. Data analysis was performed using ZetaView software (version 8.05.16 SP7), with a minimum particle size of 10, a maximum size of 1000, and a minimum brightness of 30. All measurements were conducted at room temperature (25°C). A JEOL 1230 transmission electron microscope was used for visualising sEVs. For negative staining electron microscopy, sEVs were diluted in PBS 1X, applied to copper grids, and incubated for 15 min at room temperature. Excess liquid was removed by blotting. The grids were then stained with 2% uranyl acetate (w/v) (Merck, Darmstadt, Germany) for 30 s and observed as previously described (Moyano et al. 2019).

### 2.8 Small Extracellular Vesicles Internalisation Assays

#### 2.8.1 Small Extracellular Vesicles Labelling

The uptake of sEVs by trophozoites was visualised using super‐resolution microscopy and assessed by flow cytometry after labelling the sEVs with BODIPY FL‐C5‐ceramide (Cat. No. D3521, Invitrogen), following the manufacturer's instructions, with the exception that excess dye was removed by ultracentrifugation. Briefly, enriched sEVs were suspended in 100 µL of PBS per labelling reaction, and 1 µL of a 1 mM dye stock solution was added to the samples. After mixing, the samples were incubated at 37°C for 30 min, protected from light. Unincorporated dye was removed by ultracentrifugation at 100,000 × *g* for 180 min using a 60Ti rotor (Beckman Coulter L‐70 Ultracentrifuge). The pellet containing the freshly labelled sEVs was recovered and used in uptake assays. As a control for non‐specific staining, the same procedure was applied to autoclaved sEVs.

#### 2.8.2 Small Extracellular Vesicles Uptake Assays

Trophozoites were harvested by chilling culture tubes on ice, washed three times in PBS (pH 7.4), counted using a haemocytometer, and adjusted to the required concentration. 1 × 10^5^ trophozoites were incubated with BODIPY FL‐C5‐ceramide‐labelled sEVs for 20 min, 1 h or 2 h at 37°C. A control incubation was performed with trophozoites and autoclaved labelled sEVs for 2 h at 37°C. After incubation, trophozoites were centrifuged at 700 × *g* for 15 min at 4°C. The supernatant was discarded, and the pellet was resuspended in PBS 1X. Trophozoites were placed onto poly‐L‐lysine‐coated immunofluorescence slides and incubated at 37°C for 1 h to allow cell adherence. The slides were then washed with 1× PBS, fixed with 4% paraformaldehyde for 20 min at room temperature, and washed twice. The trophozoites were then incubated with DAPI and mounted using FluoSafe (Sigma). Fluorescence was visualised using a super‐resolution confocal microscope ZEISS LSM 980 with Airyscan 2. Images were processed using Fiji software (Schindelin et al. 2012). For flow cytometry analysis, trophozoites were incubated with BODIPY FL‐C5‐ceramide‐labelled sEVs for 5 min, 20 min, 1 h, 2 h or 3 h at 37°C. A control incubation with trophozoites and autoclaved labelled sEVs was carried out for 3 h at 37°C. After incubation, trophozoites were centrifuged at 700 × *g* for 15 min at 4°C, the supernatant discarded, and the pellet resuspended in PBS 1X. The mean fluorescence intensity (MFI) was measured using a BD FACSCanto flow cytometer (BD Biosciences), and the relative distribution of 10,000 cells was analysed using FlowJo software (version 7.6.2, Tree Star, Inc., OR, USA).

### 2.9 Induction of Metronidazole‐Sensitive Phenotypes in *Giardia* Trophozoites via sEVs

Changes in the MTZ resistance fold induced by the uptake of RsEVs in wild type trophozoites were assessed after successive rounds of exposure. The concentration of RsEVs was determined using nanoparticle tracking analysis, and trophozoites were harvested and counted as described previously. Briefly, 1 × 10^1^⁰ RsEVs were incubated with 1 × 10⁷ sensitive trophozoites (cells/RsEVs ratio: 1/1000) for 2 h at 37°C in a final volume of 2 mL (PBS 1× was added as needed). After the exposure period, the contents of the tube were transferred to a 16 mL tube, supplemented with a complete growth medium, and incubated at 37°C for 24 h to allow recovery of the treated cells. This procedure was repeated for a total of four rounds. Following the four rounds of treatment, the cells were allowed to recover for 2, 5, 10 or 15 days. The MTZ IC_50_ of the treated trophozoites was determined at each recovery time point, and the corresponding resistance fold was calculated by comparing the IC_50_ of trophozoites treated with RsEVs to that of cells treated with sEVs derived from wild type strains.

### 2.10 Statistical Analyses

All statistical analyses were performed using GraphPad Prism 9 (GraphPad Software Inc., USA). Data are presented as mean ± standard error of the mean (SEM) from three independent experiments unless otherwise stated. Unpaired Student's *t*‐tests were used for comparisons between two groups, while one‐way ANOVA followed by Tukey's multiple comparisons test, was used for comparisons involving three or more groups.

## 3 Results

### 3.1 Successful In Vitro Induction of Metronidazole Resistance in Trophozoites of Genotypes A and B

**FIGURE 1:**
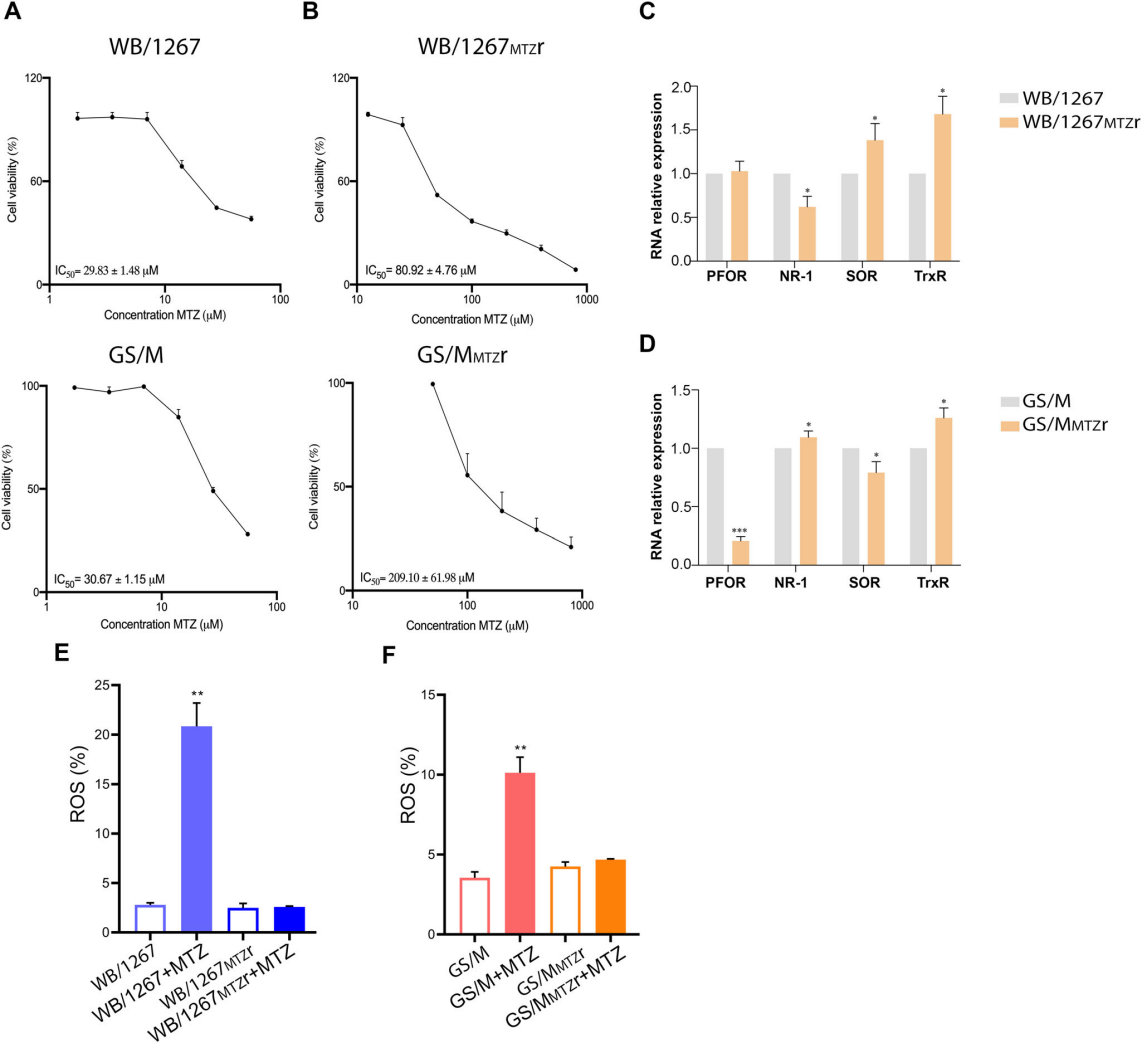
Evaluation of *Giardia lamblia* trophozoites' induced metronidazole (MTZ) resistance mechanisms. (A and B) The IC_50_ values of MTZ for induced WB/1267_MTZ_r and GS/M_MTZ_r clones are significantly higher than those of the wild type isolates WB/1267 and GS/M isolates. (C and D) Relative mRNA expression levels of enzymes implicated in MTZ activation and detoxification, including pyruvate oxidoreductase (PFOR), nitroreductase‐1 (NR‐1), superoxide reductase (SOR) and thioredoxin reductase (TrxR), measured via qPCR. (E and F) Intracellular reactive oxygen species (ROS) production in response to 20 µM MTZ was assessed using H_2_DCFDA and flow cytometry and shown as a percentage compared with the control. Mean ± SEM from three independent experiments. One‐way ANOVA followed by Tukey's post hoc test was used for multiple group comparisons. *p < 0.05; **p < 0.01; ***p < 0.001.

To produce MTZ‐resistant clones, wild type trophozoites from the WB/1267 (genotype A) and GS/M (genotype B) isolates were grown in a sublethal concentration of MTZ (see Section 2). The survival of *G. lamblia* trophozoites was evaluated in vitro by microscopic observation and the 3‐(4,5‐dimethylthiazol‐2‐yl)‐2,5‐diphenyltetrazolium bromide (MTT) colorimetric assay to determine the half‐maximal inhibitory concentrations (IC_50_) necessary to inhibit the viability of trophozoites by 50%. The IC_50_s of WB/1267 and GS/M wild type isolates were around 30 µM (Figure 1A), while the values for clones WB/1267_MTZ_r and GS/M_MTZ_r were 80 and 209 µM, respectively (Figure 1B). The resistance fold (RF = IC_50_ MTZ Resistant / IC_50_ wild type) showed a marked increase in resistance to the antiparasitic drugs studied, with RF WB/1267 MTZ = 2.7 and RF GS/M MTZ = 6.8, respectively. Except for clinical case samples, this study is the first to report laboratory‐derived MTZ‐resistant genotype B trophozoites, showing that both human‐infecting genotypes can develop resistance in vitro under suboptimal drug exposure.

### 3.2 *G. lamblia* WB/1267_MTZ_r and GS/M_MTZ_r Clones Exhibit Distinct Patterns of Enzyme Expression Involved in the MTZ's Metabolic Pathways

Due to MTZ's shallow redox potential, its metabolisation only quantitatively occurs in microaerophilic and anaerobic organisms with a strongly reductive physiology (Samuelson 1999). Resistance often entails downregulating enzymes that convert MTZ to its toxic intermediates, such as pyruvate oxidoreductase (PFOR) and Nitroreductase‐1 (NR‐1), a ferredoxin‐nitroreductase chimera as reviewed elsewhere (Thomas and Gwenin 2021; Watkins and Eckmann 2014; Leitsch et al. 2011). Conversely, resistance can be achieved by upregulating enzymes detoxifying MTZ or managing MTZ‐induced damage, such as the superoxide reductase (SOR) (Muller et al. 2013; Muller et al. 2015). Thioredoxin reductase (TrxR) shows variable regulation across resistant strains, suggesting it plays a dual role in drug activation and oxidative stress management (Leitsch et al. 2016). To investigate the transcriptional levels of these critical enzymes in WB/1267_MTZ_r and GS/M_MTZ_r clones, qPCR analysis of mRNA expression was performed for PFOR, NR‐1, SOR and TrxR, comparing these resistant lines to their susceptible wild type counterparts. The results for WB/1267_MTZ_r revealed that PFOR expression remained unchanged. In contrast, NR‐1 expression was significantly reduced (Figure 1C). Additionally, SOR expression increased compared to WB/1267 wild type (Figure 1C). Analysis of TrxR expression showed a significant increase over the susceptible wild type line (Figure 1C). These findings suggest that resistance in WB/1267_MTZ_r is due to the downregulation of NR‐1, reducing MTZ cytotoxicity, and the upregulation of SOR, which helps render MTZ inert. The increased expression of TrxR indicates a protective role in these resistant clones. For the GS/M_MTZ_r clone, a remarkable decrease in PFOR mRNA expression and a significant increase in TrxR were observed (Figure 1D). These findings may represent a combination of mechanisms involved in MTZ resistance in this clone.

MTZ reduction forms highly reactive nitro radicals, damaging essential cellular components such as DNA, proteins and lipids. Additionally, the nitro radicals react with available oxygen molecules, producing ROS. To analyse the intracellular generation of ROS in *Giardia* WB/ 1267_MTZ_r and GS/M_MTZ_r, 2′,7′‐dichlorodihydrofluorescein diacetate (H_2_DCFDA) was used and measured by flow cytometry. The results showed that after adding 20 µM of MTZ, the WB/1267_MTZ_r and GS/M_MTZ_r clones produced significantly lower levels of ROS than their wild type isogenic isolates (Figure 1E,F). These results suggest that MTZ resistance in WB/1267_MTZ_r and GS/M_MTZ_r is linked to a reduced ability to generate ROS, typically produced during the drug's activation process.

### 3.3 The Clones WB/1267_MTZ_r and GS/M_MTZ_r Show MTZ‐Resistance Stability After Discontinuation of Drug Selection

Previous studies have shown that in vitro‐generated MTZ‐resistant *G. lamblia* genotype A trophozoites can revert to drug sensitivity after encystation or excystation and after several generations without selective pressure (Tejman‐Yarden et al. 2011). However, increasing evidence suggests that clinically MTZ‐resistant trophozoites can be transmitted between patients, indicating that resistance can be a stable, transmissible phenotype (Carter et al. 2018; Requena‐Mendez et al. 2014). To explore the potential for reversion to drug sensitivity and the durability of MTZ resistance in WB/1267_MTZ_r and GS/M_MTZ_r clones, we cultured these clones for 5, 10 or 15 days without drug selection, corresponding to approximately 15, 30 and 45 generations, respectively, assuming an average doubling time of 8 h under optimal in vitro conditions (Gillin et al. 1989; Eckmann and Gillin 2001). This timeline aligns with typical MTZ treatment regimens, lasting 5–10 days with one or two doses per day (Escobedo and Cimerman 2007). MTZ IC_50_ values were calculated at each time point, showing that while resistance decreased over the 2 weeks without MTZ, the clones retained significantly higher resistance levels than their drug‐sensitive parents and original isolates (Figure S1). These experiments also establish the basis for using these clones in downstream experiments, ensuring that observed effects result from experimental variables rather than variations in baseline resistance stability.

### 3.4 MTZ‐Resistant WB/1267_MTZ_r and GS/M_MTZ_r Clones Release sEVs That Might Act as Carriers of Drug Resistance

**FIGURE 2:**
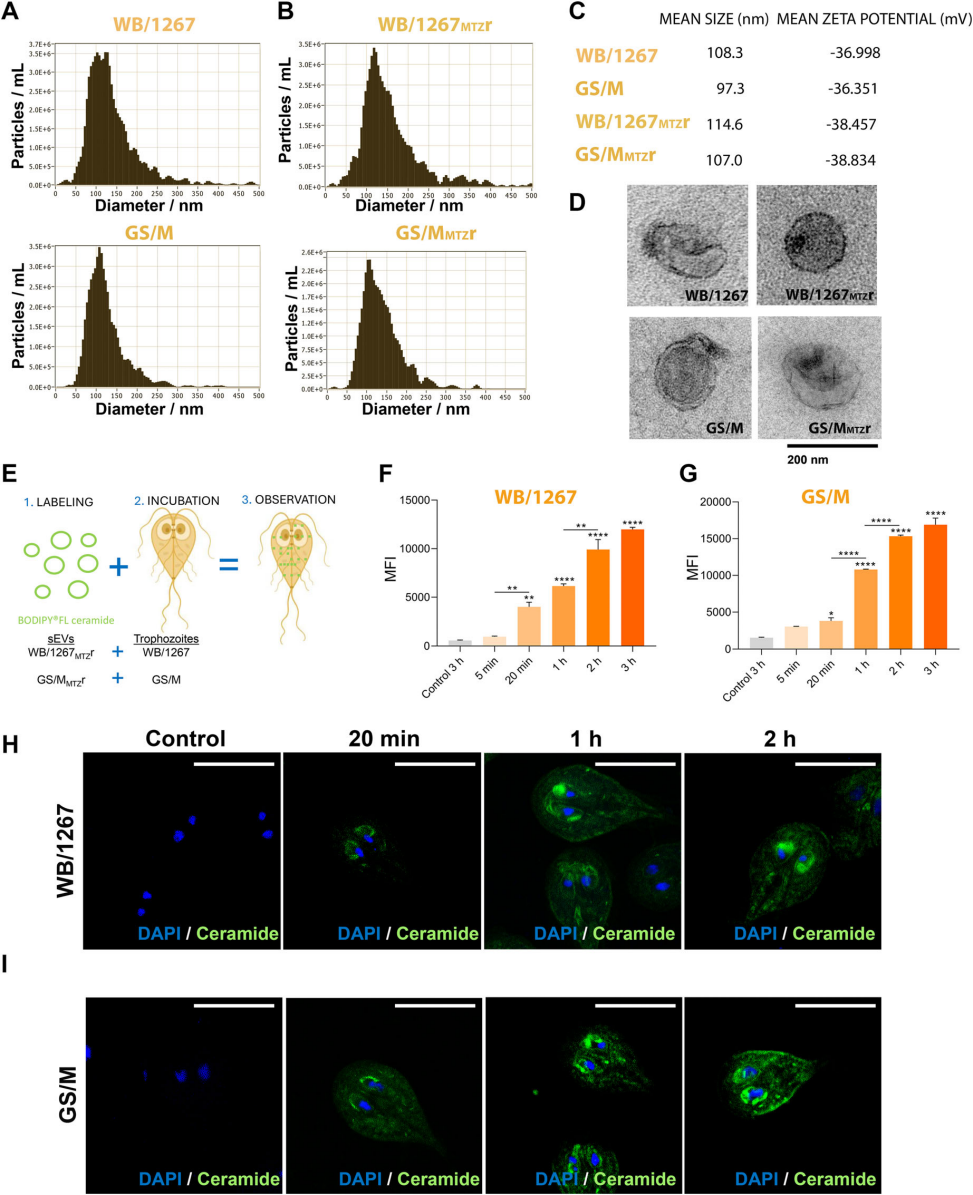
Characterisation and functional analysis of small extracellular vesicles (sEVs) released by metronidazole‐resistant (MtzR) *Giardia lamblia* clones. (A and B) Nanoparticle tracking analysis (NTA) of sEVs derived from wild type and MtzR clones WB/1267_MTZ_r and GS/M_MTZ_r. Mean particle sizes were around 100 nm for all samples, with uniform size distribution and no contaminants detected. (C) Mean particle sizes and Zeta potential (ZP) measurements of sEVs showed consistent negative values across all samples, indicating electrostatic repulsion and colloidal stability. (D) Transmission electron microscopy (TEM) revealed characteristic cup‐shaped structures with a mean diameter of approximately 100 nm. (E) Cartoon depicting the internalisation of BODIPY FL‐C5‐ceramide‐labelled sEVs from MtzR clones into wild type trophozoites. (F and G) Flow cytometry analyses of BODIPY‐associated Median Fluorescence Intensity (MFI) disclose that RsEV uptake is time‐dependent and reaches a saturation plateau after 2 h. Data are shown as mean ± SEM of three independent experiments. Statistical significance was determined by one‐way ANOVA, followed by Tukey's multiple comparisons test. *p < 0.05; **p < 0.01; ***p < 0.001; ****p < 0.0001. (H and I) Super‐resolution microscopy shows that ceramide (green) is localised to the endoplasmic reticulum (ER), perinuclear membranes (PNM) and peripheral vacuoles (PVs). Bars: 10 µm. Control experiments with disrupted sEVs or PBS showed no significant internalisation.

To investigate whether RsEVs from metronidazole‐resistant (MtzR) clones transmit resistance, we first characterised the sEV‐enriched fractions by their size distribution and zeta potential (ZP) using nanoparticle tracking analysis (NTA). The average sizes were similar across all samples, with mean diameters of 106.8 nm (Figure 2A,B). The NTA analyses also revealed that the sEV preparations were free of contaminants and uniform in size. The NTA of total extracellular vesicle preparations (including both large and small EVs) from wild‐type WB/1267 and GS/M trophozoites showed that under the conditions employed (without stimulation and lacking serum and bile), the sEVs are the predominant ones (Figure S2A,B) in both strains, suggesting that *G. lamblia* trophozoites naturally favour the release of small vesicles under physiological‐like, non‐stress conditions. We also measured the zeta potential of these sEVs, which typically range from −10 mV to −50 mV for RsEVs (exosomal) and is influenced by surface charge, affecting particle aggregation tendencies (Figure 2C) (Frohlich 2012; Filipe et al. 2010). The negative ZP values indicate that the sEVs exhibit electrostatic repulsion among particles, which helps prevent aggregation and supports colloidal stability. Transmission electron microscopy (TEM) revealed ∼100 nm, cup‐shaped sEVs across all samples (Figures 2D and S2C), consistent with previous findings (Natali et al. 2023).

*Giardia* RsEVs were tested for trophozoite communication by labelling RsEVs from WB/1267_MTZ_r and GS/M_MTZ_r clones with BODIPY FL‐C5‐ceramide, a fluorescent exosome marker in vitro and in vivo (Nicola et al. 2009). Since *Giardia* cannot synthesise ceramide de novo, it must acquire this lipid from its environment (Hernandez et al. 2007). Labelled RsEVs were washed and incubated with WB/1267 or GS/M wild type isolates (Figure 2E). Flow cytometry revealed progressive BODIPY‐ceramide uptake, which plateaued between 2 and 3 h (Figure 2F,G). Super‐resolution microscopy revealed time‐dependent ceramide incorporation into perinuclear membranes (PNM), the endoplasmic reticulum (ER) and endo‐lysosomal peripheral vacuoles (PVs) until saturation at 2 h (Figure 2H,I). Controls included incubation with disrupted RsEVs or PBS before labelling. These findings demonstrate that RsEVs from MtzR *G. lamblia* clones can be internalised by wild type trophozoites, following a pathway like that of ceramide uptake (Hernandez et al. 2007; Zamponi et al. 2017).

### 3.5 The Internalisation of RsEVs Released by Drug‐Resistant Parasites Influences the MTZ Sensitivity of Isogenic Wild Type Cells

**FIGURE 3:**
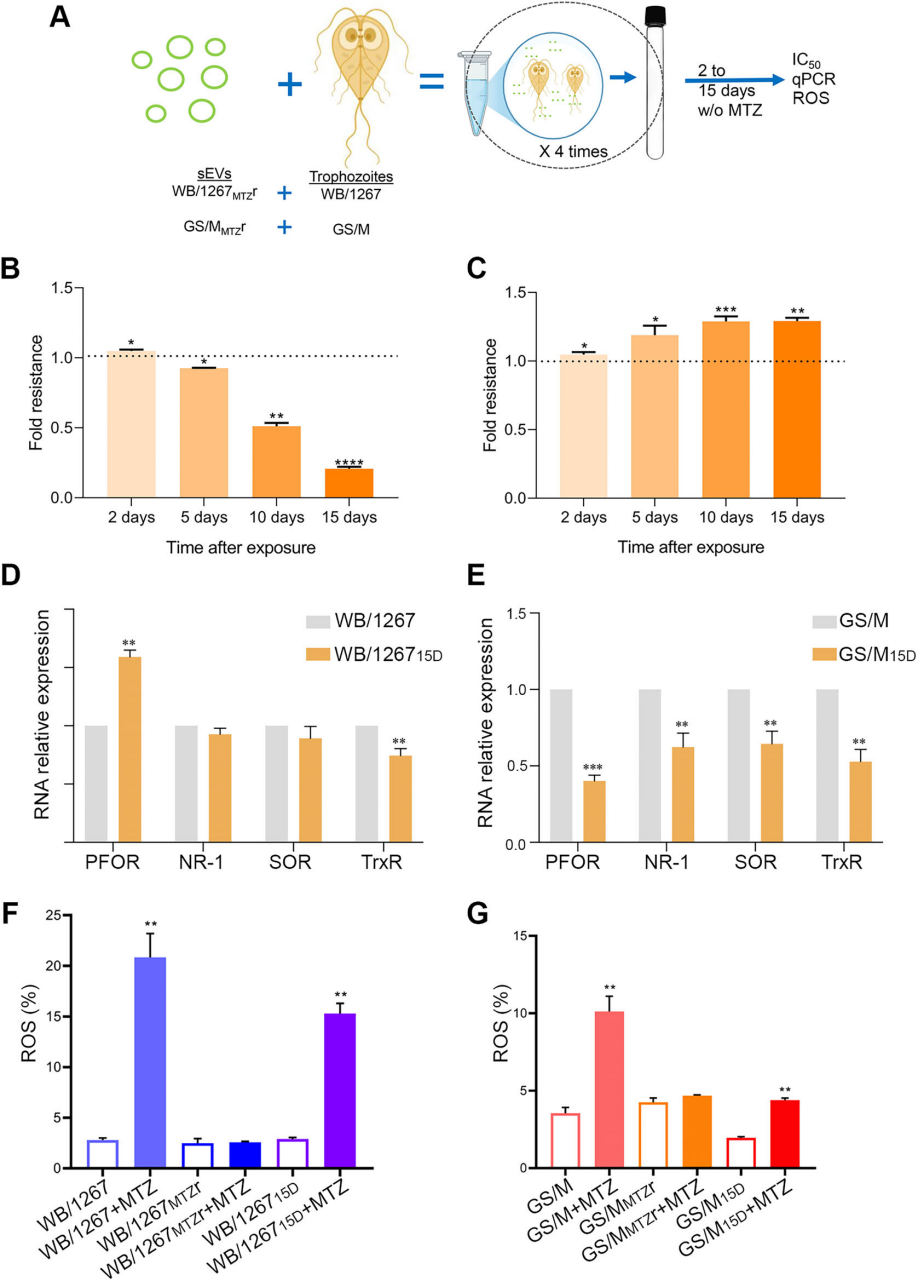
Effects of resistant small extracellular vesicles (RsEVs) on metronidazole (MTZ) sensitivity in isogenic wild type *Giardia lamblia* trophozoites. (A) Experimental optimisation of RsEV incorporation using a 1000:1 RsEV‐to‐trophozoite ratio and four cycles of 2‐h incubations with recovery periods. (B and C) Phenotypic changes in MTZ sensitivity following RsEV uptake. WB/1267 wild type trophozoites treated with RsEVs from WB/1267_MTZ_r show an ∼5‐fold increase in MTZ sensitivity by day 15 (WB/1267_15D_), while GS/M trophozoites treated with RsEVs from GS/M_MTZ_r exhibit ∼1.3‐fold decrease in MTZ sensitivity by the same time point (GS/M_15D_). Changes in IC_50_ values were compared between RsEV‐treated cells and controls. Data are presented as mean ± SEM of three independent experiments. Unpaired t‐tests were used to determine statistical significance for each time point comparison. *p < 0.05; **p < 0.01; ***p < 0.001; ****p < 0.0001. (D and E) Differential expression of MTZ metabolism‐related enzymes as assessed by qPCR. Pyruvate ferredoxin oxidoreductase (PFOR), nitroreductase‐1 (NR‐1), superoxide reductase (SOR) and thioredoxin reductase (TrxR). (F and G) Percentage of intracellular reactive oxygen species (ROS) production after treatment with 20 µM MTZ, compared to their wild type. Mean ± SEM from three independent experiments. One‐way ANOVA followed by Tukey's post hoc test was used for multiple group comparisons. *p < 0.05; **p < 0.01; ***p < 0.001; ****p < 0.0001.

After confirming RsEV transfer from MtzR to isogenic wild type trophozoites, we evaluated its impact on MTZ sensitivity. Optimal conditions were achieved with a 1000:1 RsEV‐to‐trophozoite ratio and four 2‐h incubation cycles, enhancing uptake through repeated exposures with recovery periods (see Section 2) (Figure 3A). RsEVs from WB/1267_MTZ_r initially caused an increase in the IC_50_ of MTZ in WB/1267 wild type trophozoites, indicating a transient reduction in drug sensitivity. However, after 15 days without MTZ exposure, the resulting WB/1267_15D_ cells became approximately five times more sensitive to MTZ than the untreated controls (Figure 3B). Conversely, RsEVs from GS/M_MTZ_r decreased MTZ sensitivity in GS/M wild type trophozoites by about 1.3–1.35 times after 15 days (Figure 3C). These contrasting responses between the WB and GS genotypes suggest that the impact of sEV‐mediated transfer of MTZ sensitivity traits significantly depends on the genetic background of *G. lamblia* isolates.

### 3.6 The Differential Expression of Enzymes Involved in MTZ Metabolism Might Explain the Variations in MTZ‐Sensitivity

To further investigate the differences in MTZ sensitivity, we performed qPCR to analyse the mRNA expression of PFOR, NR‐1, SOR and TrxR. WB/1267_15D_ exhibited higher PFOR and lower TrxR expression than WB/1267, suggesting increased MTZ activation and reduced detoxification, contributing to heightened sensitivity (Figure 3D). In contrast, GS/M_15D_ displayed decreased PFOR and NR‐1 expression, indicating reduced MTZ activation—an established resistance mechanism (Figure 3E). The lower expression of SOR and TrxR suggests a diminished reliance on detoxification, possibly compensated for by alternative oxidative stress responses (Figure 3E). These expression differences elucidate the contrasting MTZ responses: WB/1267_15D_ became more sensitive due to increased activation and impaired detoxification, while GS/M_15D_ lost sensitivity through reduced activation and adaptive stress mechanisms.

Since ROS production contributes to MTZ‐induced cytotoxicity, we performed the H_2_DCFDA assay on WB/1267_MTZ_r, GS/M_MTZ_r, WB/1267_15D_ and GS/M_15D_ trophozoites, comparing their ROS production to that of the respective wild type WB/1267 or GS/M genotypes. WB/1267_15D_ generated less ROS than WB/1267 but significantly more than the resistant WB/1267_MTZ_r, supporting its regained MTZ sensitivity (Figure 3F). Conversely, GS/M_15D_ showed ROS levels similar to the resistant GS/M_MTZ_r and lower than GS/M, reinforcing its lost sensitivity to MTZ (Figure 3G). These results highlight genotype‐specific MTZ sensitivity mechanisms in *G. lamblia*.

### 3.7 The Variability on MTZ‐sensitivity Transmission Might Rely on the RsEV Content and the Genetic Particularities of the Recipient Cell

The significant genetic distances between *G. lamblia* genotypes suggest they represent distinct species, supported by whole‐genome comparisons of genotypes A, B and E (Morrison et al. 2007; Franzen et al. 2009; Jerlstrom‐Hultqvist et al. 2010). While genotypes A and B cause similar gastrointestinal symptoms, genotype A is more often asymptomatic and linked to chronic infections, whereas genotype B is associated with more severe symptoms and higher recurrence rates (Andrews et al. 1992; Thompson and Lymbery 1996; Homan and Mank 2001; Sahagun et al. 2008; Haque et al. 2005). Mixed infections, reported in 2%–21% of cases (higher in economically disadvantaged regions), further complicate diagnosis (Hopkins et al. 1997; Amar et al. 2002; Traub et al. 2004; Lalle et al. 2005; Geurden et al. 2008; Puebla et al. 2014). Given that changes in MTZ sensitivity can be transferred between trophozoites, we hypothesised that sEVs might mediate this exchange across genotypes.

**FIGURE 4:**
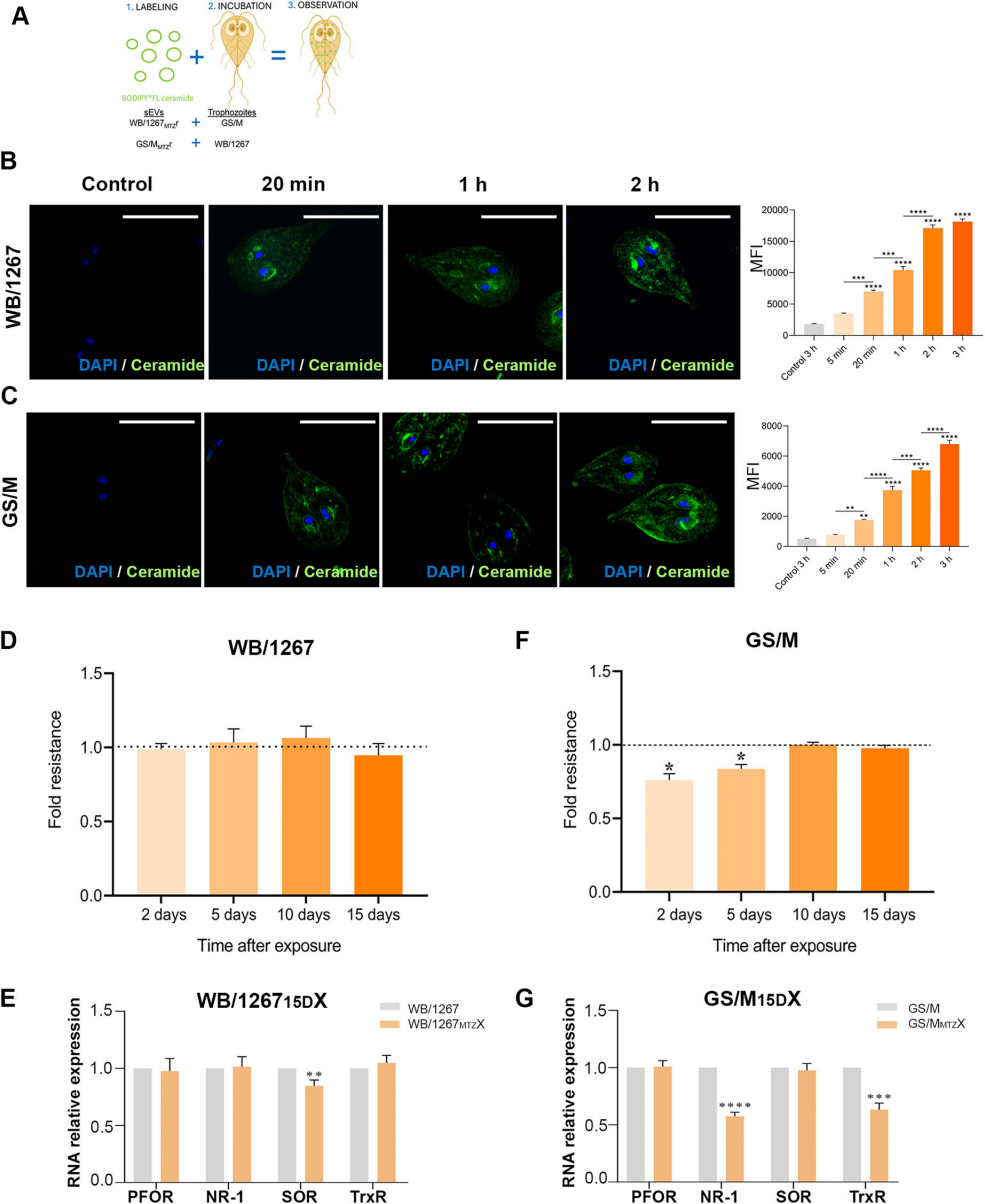
Analysis of the role of RsEVs in the transmission of metronidazole (MTZ) sensitivity between no isogenic *Giardia lamblia* genotypes. (A) Schematic representation of the experimental setup for RsEV uptake in which RsEVs from WB/1267_MTZ_r clone were incubated with the wild type GS/M isolate, while labeled RsEVs from GS/M_MTZ_r were incubated with WB/1267. (B and C) Super‐resolution microscopy images show increased RsEV uptake over time in both experimental conditions. Bars: 10 µm. The graphics of flow cytometry analyses of BODIPY‐associated Median Fluorescence Intensity (MFI) demonstrate a significant reduction in uptake when RsEVs from WB/1267_MTZ_r were incubated with GS/M trophozoites, compared to the reverse combination. (D) MTZ sensitivity was assessed by IC_50_ fold changes at different time points. No significant changes were observed when sEVs from GS/M_MTZ_r were incubated with WB/1267. (E) Enzyme expression in the derived WB/1267_15D_X strain shows no difference (except for SOR mRNA) compared to the wild type, suggesting the absence of effective resistance adaptations. (F) Increased sensitivity to MTZ (lower IC_50_) in GS/M trophozoites at early time points when incubated with sEVs from WB/1267_MTZ_r. (G) Reduced expression of NR‐1 and TrxR in the GS/M_15D_X strain indicates partial or incomplete resistance mechanisms. Pyruvate ferredoxin oxidoreductase (PFOR), nitroreductase‐1 (NR‐1), superoxide reductase (SOR), and thioredoxin reductase (TrxR). Quantitative RT‐PCR analysis of MTZ metabolism genes and ROS production in RsEV‐treated and control trophozoites. Mean ± SEM from three independent experiments. One‐way ANOVA followed by Tukey's post hoc test was used for multiple group comparisons. *p < 0.05; **p < 0.01; ***p < 0.001; ****p < 0.0001.

To test sEV uptake kinetics, BODIPY FL‐C5‐ceramide‐labeled RsEVs from WB/1267_MTZ_r were incubated with GS/M wild type trophozoites, and vice versa, following the protocol described above (Figure 4A). Super‐resolution microscopy confirmed time‐dependent RsEV uptake in both cases (Figure 4B,C). However, flow cytometry revealed significantly lower uptake when RsEVs from WB/1267_MTZ_r were incubated with GS/M trophozoites compared to the reverse scenario (Figure 4B,C, graph). Further comparison of RsEV uptake between genotypes revealed significant differences in internalisation efficiency (Figure S3A–B). Flow cytometry analysis showed that WB/1267 trophozoites internalised GS/M_MTZ_r‐derived RsEVs more efficiently than GS/M trophozoites internalised WB/1267_MTZ_r‐derived RsEVs. This asymmetric uptake suggests that genotype‐specific factors influence vesicle internalisation, which may contribute to the differential modulation of MTZ sensitivity observed in Figure 4. These results support the idea that both the origin and the recipient genotype play key roles in determining the biological impact of RsEV‐mediated communication.

When the effect of MTZ was assessed by measuring IC_50_ fold changes over time, no significant differences were observed when RsEVs from GS/M_MTZ_r were incubated with WB/1267 (Figure 4D). The resulting WB/1267_15D_X strain showed no changes in sensitivity or enzyme expression compared to the wild type, indicating a lack of adaptation (Figure 4D,E). In contrast, GS/M trophozoites incubated with RsEVs from WB/1267_MTZ_r exhibited increased sensitivity to MTZ (lower IC_50_) at early time points (Figure 4F). The GS/M_15D_X strain showed reduced NR‐1 and TrxR expression (Figure 4G), suggesting partial or incomplete loss of sensitivity mechanisms. These findings indicate that RsEVs play a crucial role in sEV‐mediated MTZ sensitivity transfer, but the recipient genotype strongly influences this process.

## 4 Discussion

This study showed, for the first time, that the sEVs in *G. lamblia* can transmit information between parasites that control their response to MTZ. Equally important, it proved that this information can be overwhelmed by features inherent to the genotype.

One approach to studying drug resistance involves using isolated strains from patients who experienced treatment failure with MTZ. However, it is challenging to distinguish between reinfection and actual drug resistance in infected patients. Moreover, isolates are complex to culture, and growth rates may differ between isolates and genotypes (Cruz et al. 2003). Consequently, the preferred method is the in vitro induction of resistance by subculturing parasites under increasing sublethal concentrations of MTZ. This approach mimics real‐world scenarios, as one cause of drug resistance is the discontinuation of treatment or suboptimal drug dose in the treatment protocol (Lalle and Hanevik 2018). Additionally, in vitro‐generated resistance clones allow for controlled experimentation and growth analysis.

Here, we showed that the WB/1267_MTZ_r and GS/M_MTZ_r clones generated in this study through prolonged in vitro exposure represent bona fide drug‐resistant lines. Their significantly elevated IC_50_ values, the stability of the phenotype after drug withdrawal, and the presence of consistent molecular changes in drug metabolism and oxidative stress pathways support this classification. Also, this study provides the first evidence of laboratory‐induced MTZ resistance in *G. lamblia* trophozoites of genotype B, specifically in the GS/M_MTZ_r clone. Starting with similar MTZ sensitivity levels as genotype A, the GS/M_MTZ_r clone developed 2.5 times greater resistance. This heightened resistance aligns with previous findings that genotype B exhibits distinct capabilities in managing oxidative stress (Saghaug et al. 2019). Furthermore, based on our experience working with both WBC6 and WB/1267 isolates of genotype A, we observed that even within the same genotype, genetic background significantly influences the pace and extent of resistance acquisition. WB/1267 showed slower growth and higher baseline MTZ tolerance compared to WBC6, which likely contributed to the longer selection period required to achieve stable resistance in our study (Garcia‐Bustos et al. 2025). These findings reinforce that inter‐genotypic and intra‐genotypic differences impact the development of drug resistance in *G. lamblia*.

Our recent findings demonstrated that *Giardia* sEVs transfer sRNAs, including shared and distinct biotypes, between trophozoites of the same genotype (Natali et al. 2023), highlighting the potential role of sEVs as carriers of specific molecules with distinct biological information. Based on previous studies linking exosomes (now called small extracellular vesicles, or sEVs) to drug resistance in tumour cells (Namee and O'Driscoll 2018; Dong et al. 2020; Lu et al. 2023; Hoshino et al. 2015), we hypothesised that sEVs could specifically be involved in transmitted adaptation to MTZ in *G. lamblia*. Recent metagenomic studies have identified antibiotic‐resistance genes within bacterial EVs, supporting these findings (Qin et al. 2022). Additionally, EVs derived from drug‐resistant *Leishmania* parasites have been shown to transfer episomal DNA containing drug‐resistance genes (Douanne et al. 2022). This transfer enables recipient parasites to exhibit enhanced growth and improved oxidative stress management. The authors of these *Leishmania* studies concluded that parasites exploit EVs—predominantly those under the 200 nm size threshold—to propagate drug‐resistance genes as part of episomal amplification (Douanne et al. 2022). This demonstrates EV‐mediated horizontal gene transfer in eukaryotic parasites and represents an alternative mechanism for drug resistance in eukaryotic cells.

Here, we demonstrated that RsEVs released by drug‐resistant parasites can efficiently alter the drug‐sensitivity phenotype of recipient parasites after 4 days of exposure, revealing a rapid adaptation and modulation of MTZ metabolism. Remarkably, the recipient trophozoites, WB/1267_15D_ and GS/M_15D_, exhibited divergent responses to RsEV‐mediated molecular exchange: WB/1267_15D_ gained sensitivity to MTZ, while GS/M_15D_ displayed loss of sensitivity. The MTZ sensitivity observed in WB/1267_15D_ trophozoites appeared to be linked to higher MTZ metabolism and reduced detoxification capacity. In contrast, GS/M_15D_ trophozoites displayed enhanced desensitisation due to decreased drug activation and altered oxidative stress response pathways. These results suggest that RsEV‐mediated molecular exchange induces rapid and genotype‐specific adaptations in *G. lamblia*, with divergent effects on drug sensitivity determined by the RsEVs information or the recipient's metabolic and stress response pathways. Moreover, the profile expression of PFOR, NR‐1, SOR and TrxR obtained from WB/1267_15D_ and GS/M_15D_, differed from the original ones shown for WB/1267_MTZ_r and GS/M_MTZ_r, respectively, suggesting that the sensitive or tolerance phenotype acquired through RsEV transfer is not identical to that generated by direct MTZ selection. Resistance induced by gradual MTZ exposure through limited dilution likely involves the direct selection and stabilisation of specific resistant phenotypes. In contrast, tolerance modulation via RsEVs appears to reflect a distinct process, where intercellular communication alters drug sensitivity without fully reproducing the resistant state. These findings suggest that extracellular vesicle‐mediated communication may drive early or partial adaptations to drug pressure, likely representing a previously underappreciated mechanism contributing to the development of drug resistance.

In our investigation of the role of RsEVs in redox control and metabolic enzyme regulation related to MTZ, we found that GS/M trophozoites incubated with RsEVs from WB/1267_MTZ_r demonstrated heightened sensitivity to MTZ at early stages, although this effect diminished after 15 days. Furthermore, the incubation of RsEVs from GS/M_MTZ_r with the wild type WB/1267 isolate did not produce significant changes. These findings indicate that the transfer of resistance through RsEVs is genotype‐dependent and not universally effective, highlighting the complexity of resistance mechanisms in these parasites.

The observed differences in MTZ‐sensitivity between GS/M and WB/1267 strains after incubation with RsEVs from isogenic clones suggest that GS/M strains possess a combination of genetic, metabolic and antioxidant factors that enhance their ability to resist the drug. These factors could include genetic mutations, higher levels of MTZ detoxifying enzymes and efficient antioxidant systems. In contrast, the increased sensitivity of WB/1267_R15_ to MTZ following exposure to RsEVs from WB/1267_MTZ_r suggests that the integration of RsEV‐derived cargo may disrupt the redox balance, alter enzyme expression, and increase ROS production, ultimately making the cells more vulnerable to MTZ. These findings highlight the complex, genotype‐specific mechanisms underlying MTZ resistance and the impact of RsEV‐mediated molecular exchange on drug sensitivity. Further investigation is needed to better understand the interplay between genetic background and sEV‐mediated resistance transfer.

Resistance to MTZ has been observed in *G. lamblia* isolates from patients and in vitro‐derived clones of genotype A. Previous studies have underscored the importance of controlling MTZ metabolism and managing the cellular response to toxic molecules, such as reactive oxygen species. However, the mechanisms underlying the acquisition of this resistance remain largely unexplored. Although poorly understood in eukaryotic parasites, epigenetic mechanisms may explain the rapid shifts in MTZ sensitivity observed when wild type trophozoites are exposed to RsEVs from isogenic or non‐isogenic clones. Emerging evidence suggests that functional crosstalk between the modification of histones and chromatin remodelling is essential in transcriptional regulation and cellular decision‐making. Our preliminary findings indicate that histone 3 modifications significantly regulate MTZ resistance in the *G. lamblia* clones studied, with distinct differences between genotypes and resistant clones (Luna Pizarro et al., results not shown). Additionally, sRNAs carried by *G. lamblia* sEVs may act as transcriptional regulators in recipient cells, as demonstrated for tsRNAs from *Trichomonas vaginalis* (Artuyants et al. 2020). Additionally, as part of our ongoing investigation into drug resistance, we are currently conducting targeted sequencing of key genes, such as PFOR and NR‐1, in both RsEV‐treated and MTZ‐resistant clones. These studies aim to determine whether stable genetic or epigenetic changes contribute to the persistence of the resistance phenotype. They will be complemented by global approaches, such as ChIP‐Seq and RNA‐Seq, to provide a more comprehensive understanding of transcriptional regulation in this context.

Since many pathogens share conserved epigenetic pathways, targeting these pathways may reveal novel drug targets, potentially leading to broad‐spectrum antiparasitic agents. The emergence of drug‐resistant microorganisms represents a growing threat to global health, particularly among immunocompromised individuals. Despite advances in prevention and diagnostics, MTZ resistance in *G. lamblia* continues to cause significant morbidity and mortality. Current therapeutic options are limited to a single primary drug, MTZ, emphasising the urgent need for alternative treatments. The availability of MTZ‐resistant *G. lamblia* clones presents an opportunity to screen already approved drugs, accelerating the discovery of new therapies to combat drug‐resistant giardiasis and other protozoan infections.

Addressing MTZ resistance in *G. lamblia* requires a multidisciplinary approach integrating molecular, epigenetic and therapeutic studies. Unravelling the mechanisms of resistance acquisition and transmission advances our understanding of giardiasis. It informs the broader fight against drug‐resistant pathogens, underscoring the critical need for innovation in antiparasitic drug development.

## Author Contributions

**Gabriel Luna Pizarro**: conceptualization, investigation, methodology, formal analysis. **Jerónimo Laiolo**: investigation, formal analysis, conceptualization, writing ‐ review and editing, funding acquisition. **Nehuén Salas**: methodology. **Rocío G. Patolsky**: methodology. **Luciano Díaz Pérez**: methodology. **Camilo Cotelo**: methodology. **Constanza Feliziani**: investigation, writing ‐ review and editing, supervision. **Andrea Silvana Rópolo**: investigation, writing ‐ review and editing, funding acquisition. **María Carolina Touz**: conceptualization, investigation, funding acquisition, writing ‐ original draft, writing ‐ review and editing, validation, supervision, resources, project administration, visualization.

## Conflicts of Interest

The authors declare no conflicts of interest.

## Declaration of Generative AI and AI‐Assisted Technologies in the Writing Process

During the preparation of this work, the author(s) utilized ChatGPT (OpenAI, 2024) and *Grammarly* (https://www.grammarly.com) to assist with English language refinement and readability. The author(s) carefully reviewed and edited all content as necessary and assume(s) full responsibility for the final version of the publication. Schemes for the figures were created using BioRender.com.

## Additional Information

Further information and requests for resources and reagents should be directed to and will be fulfilled by the lead contact, María Carolina Touz (ctouz@immf.uncor.edu).

## Supporting information




**Supplementary Fig.1**: jev270139‐sup‐0001‐figureS1.pdf

**Supplementary Fig.2**: jev270139‐sup‐0002‐figureS2.pdf

**Supplementary Fig.3**: jev270139‐sup‐0003‐figureS3.pdf

## Data Availability

The data that support the findings of this study are available from the corresponding author upon reasonable request.
