# Supplementary material for: Genotype‐Specific Small EVs Released by Giardia lamblia Act as Mediators of Phenotypic Adaptation Under Metronidazole‐Induced Stress
Source: J Extracell Vesicles. 2025 Sep 1;14(9):e70139. doi: 10.1002/jev2.70139 (PMC12399883; doi:10.1002/jev2.70139)
Supplement: Supplementary file 1 — Supplementary Fig.1: jev270139‐sup‐0001‐figureS1.pdf [file JEV2-14-e70139-s001.pdf]

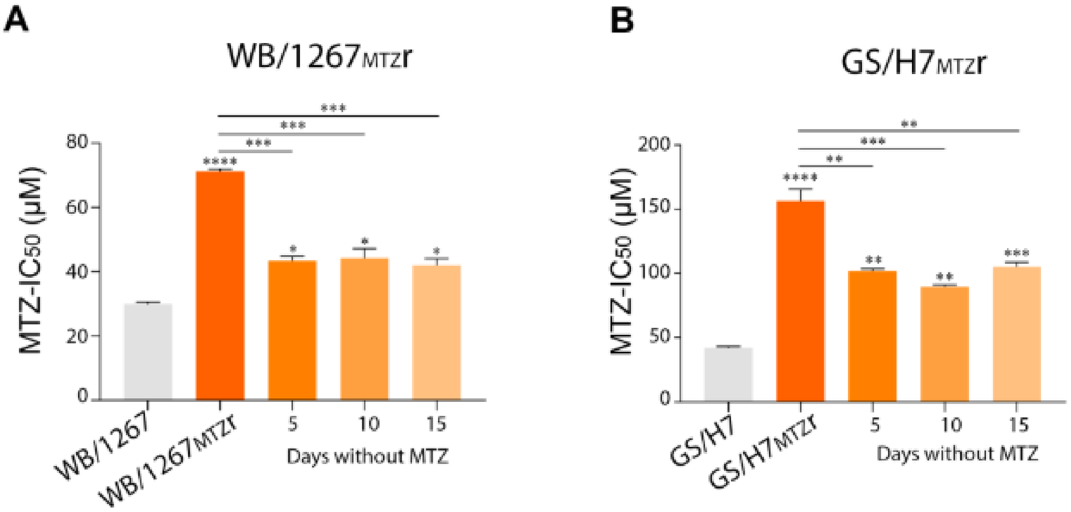

Supplementary Figure 1: **Assessment of metronidazole (MTZ) resistance stability in *Giardia lamblia* clones WB/1267$_{MTZ}$r and GS/M$_{MTZ}$r during culture without drug selection.** MTZ-resistant clones were cultured for 5, 10, and 15 days without MTZ to evaluate the potential for reversion to drug sensitivity. IC_{50} values for MTZ were calculated at each time point. Significance was determined by comparing the clones cultured without the drug and the wild-type cells (* over each bar) and between the MTZr cells and the drug-free culture lines (* over the lines). Statistical significance levels are: *p < 0.05; **p < 0.01, ***p < 0.001.
