# Supplementary material for: Genotype‐Specific Small EVs Released by Giardia lamblia Act as Mediators of Phenotypic Adaptation Under Metronidazole‐Induced Stress
Source: J Extracell Vesicles. 2025 Sep 1;14(9):e70139. doi: 10.1002/jev2.70139 (PMC12399883; doi:10.1002/jev2.70139)
Supplement: Supplementary file 2 — Supplementary Fig.2: jev270139‐sup‐0002‐figureS2.pdf [file JEV2-14-e70139-s002.pdf]

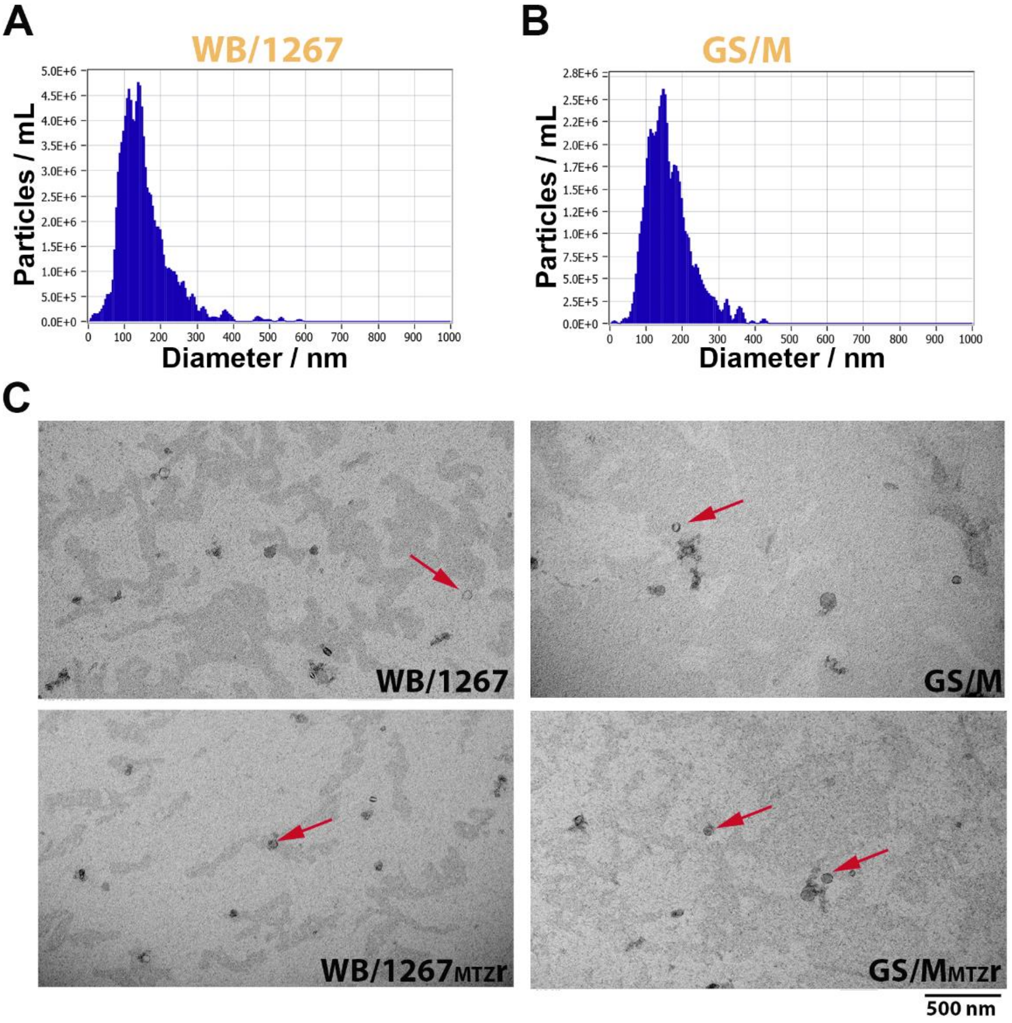

Figure S2. Wild type and MTZ-resistant *Giardia lamblia* trophozoites release small extracellular vesicles (sEVs). (A–B) Nanoparticle tracking analysis (NTA) of total extracellular vesicle preparations (including both large and small EVs) from wild type WB/1267 and GS/M trophozoites. For WB/1267, 53.3% of particles measured 138.5 nm and 39.7% measured 113.3 nm. For GS/M, 96.5% of particles were 144.9 nm and 3.5% were 319.5 nm, indicating a strong enrichment in small vesicles in both strains. (C) Transmission electron microscopy (TEM) image of vesicles obtained after sEV-specific purification, showing characteristic cup-shaped structures (e.g. red arrows) with a mean diameter of approximately 100 nm, a uniform size distribution, and no visible contaminants. Despite differences in the preparation protocols, both analyses consistently show a predominant enrichment of small EVs in both *Giardia* genotypes.
