# Supplementary material for: Genotype‐Specific Small EVs Released by Giardia lamblia Act as Mediators of Phenotypic Adaptation Under Metronidazole‐Induced Stress
Source: J Extracell Vesicles. 2025 Sep 1;14(9):e70139. doi: 10.1002/jev2.70139 (PMC12399883; doi:10.1002/jev2.70139)
Supplement: Supplementary file 3 — Supplementary Fig.3: jev270139‐sup‐0003‐figureS3.pdf [file JEV2-14-e70139-s003.pdf]

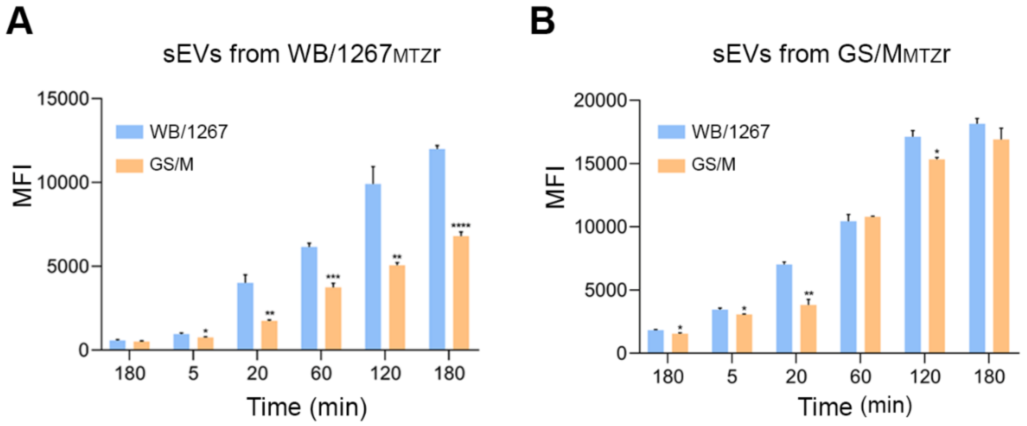

Figure S3: Analysis of variations in RsEV uptake by genotype. (A-B) Flow cytometry analysis of BODIPY-associated Median Fluorescence Intensity (MFI) reveals a significant reduction in RsEV uptake when vesicles derived from WB/1267$_{MTZ}$r trophozoites are incubated with GS/M trophozoites, compared to the reverse combination. Statistical analysis was performed using one-way analysis of variance (ANOVA). *p < 0.05; **p < 0.01, ***p < 0.001, ****p < 0.0001.
